# Public awareness of microplastic pollution among residents of a western Turkish province and how it is reflected in demographics and behavioural practices

**DOI:** 10.2478/aiht-2026-77-4030

**Published:** 2026-03-30

**Authors:** Ömer Faruk Tekin, İnci Arıkan, Muhammet Ali Bölükbaş

**Affiliations:** Kütahya Health Sciences University Faculty of Medicine, Department of Public Health, Kütahya, Turkey

**Keywords:** age differences, gender differences, education, environmental health, plastic, socioeconomic factors, dobne razlike, okolišno zdravlje, obrazovanje, plastika, rodne razlike, socioekonomski čimbenici

## Abstract

This cross-sectional study aimed to evaluate the public awareness of and attitudes toward microplastic pollution in the Kütahya Province of Turkey between September and December 2024 and included 406 users of regional family healthcare centres. Data were collected with a specifically developed questionnaire combining sociodemographic information and self-reported knowledge, attitudes, and behaviour related to the microplastics issue and with our Microplastic Pollution Awareness Scale (MPAS), against which we compared the answers to the first questionnaire. MPAS scores were significantly higher among women than men, persons under the age of 40, unmarried participants, university graduates (5.1 times higher than in those with lower education), and residents of developed regions (2.3 times higher than among residents of underdeveloped or developing regions). Overall, participants show pretty good awareness of microplastic pollution. Future prospective studies should cover all ages, but more importantly, develop effective intervention programmes aimed at changing public behaviour.

## Abstract (hr)

Ovo presječno ispitivanje imalo je za cilj procijeniti razinu javne svijesti i stavova o onečišćenju mikroplastikom u pokrajini Kütahya u Turskoj u razdoblju od rujna do prosinca 2024., a obuhvatilo je 406 korisnika područnih centara obiteljske zdravstvene skrbi. Podaci su prikupljeni pomoću za tu priliku posebno osmišljenog upitnika, koji obuhvaća sociodemografske podatke te samoprocijenjeno znanje, stavove i ponašanja povezana s mikroplastikom te pomoću naše Ljestvice svjesnosti o onečišćenju mikroplastikom (engl. *Microplastic Pollution Awareness Scale*, krat. MPAS), s kojom smo usporedili odgovore iz prvog upitnika. Rezultati na ljestvici bili su značajno viši u žena nego muškaraca, u osoba mlađih od 40 godina, neoženjenih, osoba sa završenim sveučilišnim obrazovanjem (5,1 puta viši nego u ispitanika s nižom razinom obrazovanja) te u stanovnika razvijenih područja (2,3 puta viši nego u stanovnika slabije razvijenih područja). Općenito, sudionici su pokazali prilično dobru razinu svijesti o onečišćenju mikroplastikom. Buduća prospektivna istraživanja trebala bi obuhvatiti sve dobne skupine i, što je još važnije, osmisliti učinkovite intervencijske programe, usmjerene na promjenu ponašanja javnosti.

Microplastics are tiny plastic particles, usually up to 5 mm in diameter, either present in personal care products such as sunscreens, cleansers, and cosmetics (1,2,3,4,5,6,7,8) or a result of fragmentation/degradation of larger plastic items by ultraviolet radiation, oxidation, or mechanical abrasion (7,8,9,10) (Table 1).

**Table 1:** The size ranges, types, and sources of microplastics

**Sources**	Primary: plastic products, accidental losses, surface run-off, and industrial abrasives. Microbeads, pellets, microfibers, and tire dust. Secondary: degraded plastics (from bottles, bags, packaging, paint coating, and abrasive materials) released from waste depots, landfills, and recycling facilities.
**Usage areas**	Individual consumers: cosmetics and personal care products, plastic bags, containers, bottles, caps, cups, plates, spoons, and straws. Industry consumers: raw materials, construction, packaging, agriculture greenhouse-sheets, pots, pipes, nutrient prills, textile products, and terrestrial transportation (tires and tire dust). Producers/converters: plastic producers and recyclers. Entry points into the ocean: water and wastewater, rivers, coastline, and atmosphere.
**Shapes**	Fibres, fragments, beads, foams, films, pellets, granular plastics.
**Composition**	Polyethylene (packaging), polystyrene (foam products), polypropylene (containers and ropes), polyester (textiles), polyamide (fabrics), and polyethylene terephthalate (PET, bottles and packaging).
**Size (diameter)**	25 mm–5 mm (mesoplastics); ≤5 mm–1 mm (microplastics), <1 mm–1 µm (mini-microplastics); <1 µm (nanoplastics).

More than 450 million tonnes of plastic is produced worldwide each year, and this amount is expected to increase (8, 11). Environmental exposure to microplastics includes airborne dust particles, food, and beverages (8, 9, 11) and can lead to serious adverse effects (12, 13), including systemic diseases in humans (14,15,16,17,18,19,20,21,22,23,24,25,26,27,28,29,30).

Raising awareness about the devastating effects of plastic waste pollution on the environment and society can help to reduce microplastic pollution on individual and systemic levels. Several studies have therefore tried to establish the level of public awareness about this issue and obstacles to reduce the microplastic burden (6, 23,24,25,26,27,28,29,30). We believe, however, that local or regional studies of the issue can be even more useful, as they can inform collaborative action involving communities and decision-makers (28). The policies and strategies implemented in Turkey primarily focus on reuse and recycling of waste. In this context and in line with the Sustainable Development Goals, the Zero Waste Project was launched in our country in 2017. It is a waste management system that aims to minimise waste generation and maximise recycling (31). While it is systematically integrated into official institutions, it can sometimes be difficult for the general public to comply. In addition, it is necessary for the public of our region to be aware not only of the plastic but also of the microplastic threat.

There are studies in Turkey where microplastic pollution awareness is evaluated in specific groups such as university students and biology teachers (32,33,34,35,36), whose awareness is expected to be high, but we believe it is more important to determine the level of awareness across all segments of society.

## PARTICIPANTS AND METHODS

This cross-sectional study was carried out between September and December 2024 in the Kütahya province in western Turkey with the population of 571,000, 276,000 of which is urban. Of the six waste recycling facilities in our province, three recycle plastic waste.

The minimum sample size for the study was determined to be 384 with a 95 % confidence interval, 5 % margin of error, and 50 % prevalence (cases with unknown prevalence). The participants were recruited from six family health centres, two from each of the three regions of the central district of Kütahya: one underdeveloped, one developing, and one developed (by socioeconomic standards).

Seventy people were invited from each family health centre via random sampling. The inclusion criteria were age 18–65 years, being literate, having no cognitive impairment, and completing more than 80 % of the questionnaire. Of the 442 people who agreed to participate, 36 did not complete the required 80 % of the questionnaire, and our final sample was 406 participants. All were briefed on the study and signed informed consent.

The study followed the Declaration of Helsinki and was approved by the Kütahya Health Sciences University Non-Interventional Ethics Committee (approval No. 2024/13-07).

Our study was designed to assess knowledge and individual practices regarding microplastics pollution and management with a questionnaire and to compare these data with an awareness scale. Through multiple-choice format the questionnaire gathers participants’ sociodemographic data, self-reported knowledge about the sources of microplastics, and their attitudes and behaviours related to microplastic pollution.

The 14-item Likert-type Microplastic Pollution Awareness Scale (MPAS) developed by Güleşir et al. in 2022 (32) was used to rate their awareness about prevention of microplastic pollution, effects of microplastic pollution on living beings, and effects of microplastic pollution on human health. Each item on the scale is answered as “No” (0 points), “Not sure” (1 point), or “Yes” (2 points)”. The maximum score is 28, and higher score means higher level of awareness. The reliability coefficient of the scale is 0.81.

Independent variables were participants’ sociodemographic characteristics and self-reported knowledge and behavioural practices regarding microplastic pollution.

The dependent variables were the MPAS score and microplastic pollution awareness status [a categorical variable derived from the evaluation of MPAS score using receiver operating characteristic (ROC) analysis]. The microplastic pollution awareness level of participants who scored 22 or above on the scale was evaluated as sufficient.

### Statistical analysis

The obtained data were analysed using the SPSS Statistics for Windows, version 25.0 (IBM Corp., Armonk, NY, USA). Descriptive statistics are presented as means and standard deviations or medians and ranges for numerical values and as numbers and percentages for categorical and/or nominal variables. The Mann-Whitney *U* and the Kruskal-Wallis tests were used to compare numerical variables that were not normally distributed. The groups for which we established significance with the Kruskal-Wallis test underwent Bonferroni correction.

The answers given to the question “How do you evaluate your level of knowledge about microplastics?” were used as the gold standard to determine the predictive power of the scale. The sensitivity, specificity, and area under the curve were calculated with the ROC curve by assuming self-reported moderate or better knowledge as the cut-off.

For further analysis we used two logistic regression models for variables with a p value of <0.10 obtained with univariate analyses. To select variables we relied on the backward elimination method, and the Hosmer-Lemeshow test was used to evaluate the goodness of fit of the models. Statistical significance was set to p<0.05.

## RESULTS AND DISCUSSION

**Figure 1:**
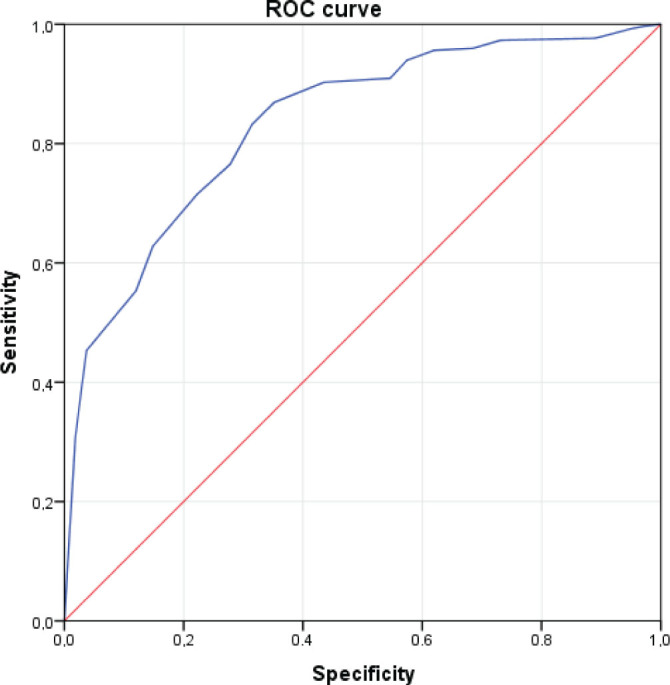
ROC curve illustrating the predictive value of the scale

ROC analysis revealed 83 % sensitivity and 69 % specificity with a cut-off point of 21.5 (AUC=0.835, 95 % CI: 0.792–0.877, p<0.001) (Figure 1).

Even though 74 % of the participants reported that they knew about the microplastic pollution in the questionnaire, 69.5 % actually scored ≥22 points on the MPAS, which was the threshold for “sufficient” awareness of this issue.

**Figure 2:**
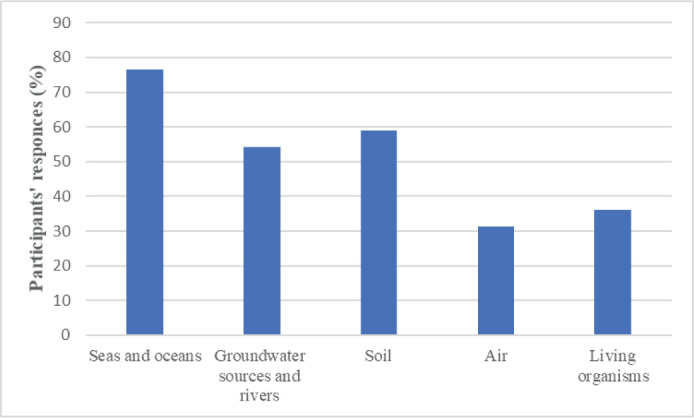
How many participants find that microplastics is present in living organisms, air, soil, and water (%)

**Figure 3:**
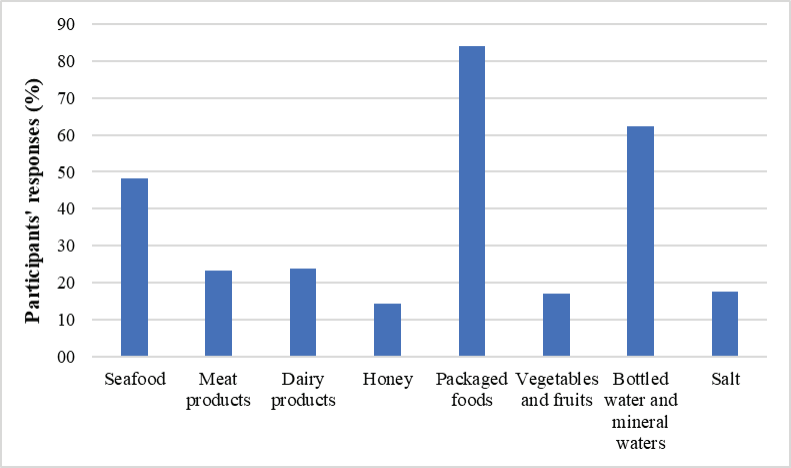
How many participants find that microplastics is present in food and drinking water

**Figure 4:**
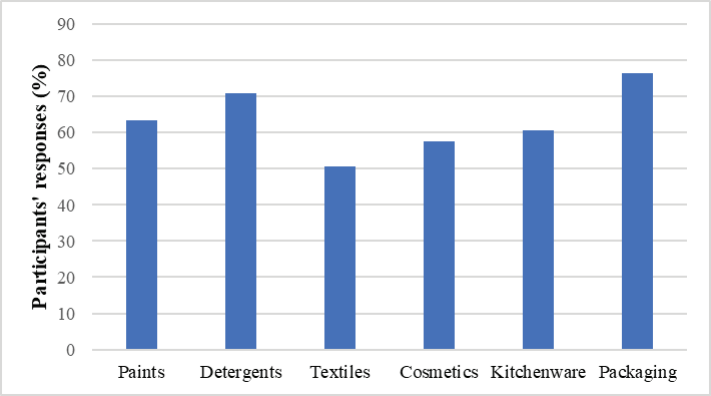
How many participants find that microplastics is present in household products

Figures 2–4 show the distribution of answers to the questionnaire regarding the presence of microplastics in the environment, foods and beverages, and household products.

Table 2 shows the participants’ demographics and MPAS scores by demographic groups. Women, participants under the age of 40, unmarried participants, participants with university or higher education, and those with high income had significantly higher MPAS scores than their counterparts.

The higher MPAS score among women in our study is quite in line with earlier reports, such as that of Yusuf et al. (6) or the gender study on environmental awareness across 22 countries (37). We can only speculate whether this is owed to the fact that women more often use cleaning, personal care, and kitchen products in daily life. In contrast, earlier studies in Turkey among university students found no significant gender differences in this respect (32, 35).

As for age differences, participants younger than 40 showed higher microplastic pollution awareness than the group aged 40–65. Since older people have more life experience and have observed a decline in environmental quality, one would expect greater environmental awareness (6, 27, 38), but young people seem to take the advantages of digital platforms and social media (39).

Table 3 shows significantly higher MPAS scores in participants answering that microplastics were present in the environment, those who disposed of waste in accordance with recycling guidelines, those who reported low, moderate, or high level of knowledge about microplastics than those who reported no knowledge on the subject, those who believed that microplastics could accumulate in the human body, those who did not believe that detergent and cosmetic product microplastic residues could be removed with water, those who refrained from heating food in plastic containers in a microwave, those who consulted the list of ingredients on the packaging before purchase, and those who believed that microplastics could enter breast milk.

More than 70 % of the participants answered there were microplastics in sea and soil environments detergents, packaging, and paints, food and beverages, mostly in those packaged in plastic. In addition, 88 % thought that microplastics could accumulate in the human body, which points to a much higher awareness than in Malaysia (6, 26) or Shanghai, China (27).

The Hosmer-Lemeshow test results for logistic regression confirmed a good fit (p=0.769) for Model 1, in which the MPAS score ≥22 (categorised as “sufficient awareness”) was 5.1 times (p<0.001) more frequent among participants with university education and above than in those with lower education level and 2.3 times (p=0.040) more frequent in those living in developed regions than in those living in underdeveloped or developing ones (Table 4). Significantly higher microplastics pollution awareness among participants with university education was an expected outcome in line with the above referenced report from India (39).

**Table 2:** Comparison of the Microplastic Pollution Awareness Scale (MPAS) scores by sociodemographic characteristics

	**Number (Percentage)**	**MPAS score**		**Statistical analysis (Z/H; p)**

		**Average ± SD**	**Median (Min–Max)**	
**Gender**				

Women	237 (58.3)	23.78±4.51	25 (11–28)	−2.656;
Men	169 (41.7)	22.45±4.98	24 (11–28)	0.008^*^

**Age**				

Under 40	239 (58.9)	23.70±4.64	25 (12–28)	−2.795;
40 and above	167 (41.1)	22.43±4.89	23 (11–28)	0.005^*^

**Marital status**				

Married	249 (61.4)	22.59±5.00	24 (11–28)	−3.001;
Unmarried	157 (38.6)	24.10±4.29	26 (12–28)	0.003^*^

**Education**				

Primary school and below	86 (21.2)	20.60±5.04	21 (11–28)	70.428;
Secondary school	107 (26.4)	21.64±4.83	22 (11–28)	<0.001^*^
University and above ^**^	213 (52.5)	25.00±3.81	27 (12–28)	

**Income** (N=397)				

Low	91 (22.9)	22.85±4.86	24 (11–28)	11.620;
Moderate	212 (53.4)	22.69±4.96	25 (11–28)	0.003^*^
High ^**^	94 (23.7)	24.72±3.77	26 (13–28)	

**Working status**				

Employed	206 (50.7)	23.13±4.83	24 (11–28)	−0.284;
Unemployed	200 (49.3)	23.24±4.74	25 (11–28)	0.777

**Residence**				

Rural	39 (9.6)	21.95±5.20	24 (11–28)	−1.645;
Urban	367 (90.4)	23.36±4.67	25(11–28)	0.100

**Socioeconomic level of the place of residence**				

Developed	63 (15.5)	24.94±4.06	26 (11–28)	
Developing	175 (43.1)	23.50±4.60	25 (11–28)	17.221;
Underdeveloped^**^	168 (41.4)	22.19±4.99	23 (11–28)	<0.001^*^

* significant difference (p<0.05).

** significant difference from this group (p<0.05).

H – Kruskal-Wallis test; Z – Mann-Whitney *U* test

Good fit (p=0.372) was also determined with the Hosmer-Lemeshow test results for logistic regression Model 2, where “sufficient” microplastic pollution awareness (MPAS score ≥22) was 2.3 times (p=0.001) more frequent in those who disposed of waste appropriately for recycling, 2.6 times (p=0.010) more frequent in those who thought that microplastics could accumulate in the human body, 1.9 times (p*=*0.021) more frequent in those who did not think that residues of detergent and cosmetic products were eliminated with water, 2.8 times (p=0.001) more frequent in those who did not heat food in plastic storage containers, and 2 times (p=0.008) more frequent in those who thought that microplastics could pass into breast milk (Table 5).

## CONCLUSION

Our main finding is that 69.5 % of the study participants actually showed sufficient awareness of microplastic pollution, which is a good level of public awareness in the western province of Turkey. We have also learned that this awareness is reflected in sociodemographic and behavioural differences in our population sample. The microplastic pollution awareness score was higher in women, those with high education, and those with high income. It was also higher in participants who reported waste recycling and avoiding heating food in plastic container and who reported that microplastics can accumulate in the human body and that residues of detergent and cosmetic products cannot be removed with water.

The main limitation of our study is that it did not include participants over 65 years of age and therefore does not cover an entire population. Future studies should take this into account, but more importantly, develop effective intervention programmes aimed at changing public behaviour. They should therefore assess baseline awareness/knowledge/behaviour and repeat the measurements after the intervention programmes have been completed in the form of prospective studies, including later-date follow-ups. They should also encompass different regions of Turkey and other countries to examine regional differences.

**Table 3:** Comparison of the Microplastic Pollution Awareness Scale (MPAS) score by self-reported knowledge and behavioural characteristics about the environment and microplastics

	**Number (%)**	**MPAS score**		**Statistical analysis (Z/H; p)**

		**Average ± SD**	**Median (Min–Max)**	
**Environmental awareness**				

Yes	377 (92.9)	23.45±4.66	25 (11–28)	−3.131;
No	29 (7.1)	20.20±5.08	20 (11–28)	0.002^*^

**The state of reacting to those who are insensitive to the environment**				
Never ^**^	37 (9.1)	21.51±5.08	22 (12–28)	7.597; 0.059
Sometimes	233 (57.4)	23.10±4.79	24 (11–28)	
Often ^**^	82 (20.2)	24.10±4.47	25.5 (11–28)	
Always	54 (13.3)	23.55±4.53	25 (13–28)	

**Disposing of garbage in a recyclable manner and knowing the recycling symbol**				

Yes	289 (71.2)	23.67±4.74	25 (11–28)	−3.667;
No	117 (28.8)	21.97±4.67	22 (11–28)	<0.001^*^

**Participants’ level of knowledge about MPs according to their own statements**				

Low level	105 (25.9)	23.05±5.01	25 (11–28)	
Moderate level	164 (40.4)	24.69±3.80	26 (11–28)	68.297;
Good level	34 (8.4)	25.56±3.81	27 (13–28)	<0.001^*^
Do not know ^**^	103 (25.4)	20.13±4.73	20 (11–28)	

**Thinking that microplastics can accumulate in the human body**				

Yes	359 (88.4)	23.77±4.47	25 (11–28)	−5.923;
No	47 (11.6)	19.11±4.86	19 (11–28)	<0.001^*^

**Thinking that residues from detergents and cosmetics are removed with water**				

Yes	93 (22.9)	21.10±5.00	22 (11–28)	−5.045;
No	313 (77.1)	23.82±4.52	25 (12–28)	<0.001^*^

**Using plastic kitchenware**				

Yes	382 (94.1)	23.13±4.82	24 (11–28)	−0.436;
No	24 (5.9)	24.00±3.99	25 (14–28)	0.663

**Heating food in plastic storage containers in the microwave**				

Yes	59 (14.5)	21.74±4.42	21 (14–28)	−2.829;
No	347 (85.5)	23.45±4.79	25 (11–28)	0.005^*^

**Frequency of consuming seafood**				

Rarely	106 (26.1)	22.91±5.11	25 (11–28)	
Occasionally	229 (56.4)	23.22±4.55	24 (11–28)	0.674;
Frequently	71 (17.5)	23.46±5.01	25 (12–28)	0.714

**Frequency of consuming processed and/or packaged foods**				

Rarely	120 (29.5)	22.97±4.64	24 (11–28)	1.253;
Occasionally	170 (42.0)	23.27±4.76	24 (11–28)	0.535
Frequently	116 (28.5)	23.36±4.86	25 (12–28)	

**Reading the ingredients of purchased products**				

Yes	226 (55.7)	23.28±4.78	25 (11–28)	−0.114;
No	180 (44.3)	23.17±4.68	24 (11–28)	0.909

**To refrain from purchasing a product based on the contents specified in the ingredients**				

Yes	246 (60.6)	23.68±4.61	25 (12–28)	−2.510;
No	160 (39.4)	22.48±4.88	23 (11–28)	0.012^*^

**Use of plastic bottles or demijohns as drinking water**				

Yes	316 (77.8)	23.32±4.73	25 (11–28)	−0.975;
No	90 (22.2)	22.83±4.81	24 (11–28)	0.330

**Leaving drinking water in plastic bottles under the sun for a long time**				

Yes	48 (11.8)	22.04±5.56	24 (11–28)	−1.192;
No	358 (88.2)	23.35±4.65	25 (11–28)	0.233

**Use of paper/cardboard cups**				

Yes	280 (69.0)	23.20±4.83	24 (11–28)	−0.416;
No	126 (31.0)	23.13±4.68	25 (11–28)	0.677

**Tea bag consumption**				

Yes	203 (50.0)	23.38±4.81	25 (11–28)	−1.098;
No	203 (50.0)	23.04±4.68	24 (11–28)	0.272

**Use of plastic feeding bottles/plastic water bottles for your child** (N=379)				

Yes	152 (40.1)	22.68±4.68	23.5 (11–28)	−1.569;
No	227 (59.9)	23.36±4.83	25 (11–28)	0.117

**Thinking that microplastics can pass into breast mi**lk (N=396)				

Yes	290 (73.2)	23.97±4.48	26 (11–28)	−5.155;
No	106 (26.8)	21.33±4.84	22 (11–28)	<0.001^*^

* significant difference (p<0.05).

** significant difference from this group (p<0.05).

H – Kruskal-Wallis test; Z: – Mann-Whitney *U* test

**Table 4:** Multiple logistic regression analysis of self-reported microplastic knowledge^*^ and some sociodemographic variables (Model 1)

**Sociodemographic variables**		**OR**	**95 % CI for OR**		**p**

			**Lower**	**Upper**	
**Gender**	Man	1			
	Women	1.576	0.987	2.516	0.057

**Educational status**	Primary school and below	1			
	Secondary school	1.498	0.827	2.712	0.183
	University and above	5.067	2.844	9.028	**<0.001**

**Development level of the place of residence**	Low	1			
	Medium	1.388	0.857	2.248	0.182
	High	2.345	1.042	5.277	**0.040**

* Categorical variable: MPAS score 22 or above on the scale was considered sufficient and scoring below 22 insufficient awareness. CI – confidence interval; OR – odds ratio

**Table 5:** Multiple logistic regression analysis of self-reported microplastic knowledge^*^ and attitudes and behaviour related to microplastics (Model 2)

**Attitudes and behaviour**		**OR**	**95 % CI for OR**		**p**

			**Lower**	**Upper**	
**Disposing of garbage in a recyclable manner and knowing the recycling symbol**	No	1			
	Yes	2.294	1.392	3.781	0.001

**Thinking that microplastics can accumulate in the human body**	No	1			
	Yes	2.574	1.249	5.303	0.010

**Thinking that residues from detergents and cosmetics are removed with water**	Yes	1			
	No	1.907	1.103	3.297	0.021

**Heating food in plastic storage containers in the microwave**	Yes	1			
	No	2.826	1.503	5.313	0.001

**Thinking that microplastics can pass into breast milk**	No	1			
	Yes	2.026	1.200	3.422	0.008

* Categorical variable: MPAS score 22 or above on the scale was considered sufficient and scoring below 22 insufficient awareness. CI – confidence interval; OR – odds ratio
